# Biogas Upgrading Using a Single-Membrane System: A Review

**DOI:** 10.3390/membranes14040080

**Published:** 2024-03-27

**Authors:** Wirginia Tomczak, Marek Gryta, Monika Daniluk, Sławomir Żak

**Affiliations:** 1 Faculty of Chemical Technology and Engineering, Bydgoszcz University of Science and Technology, ul. Seminaryjna 3, 85-326 Bydgoszcz, Poland; monika.daniluk@pbs.edu.pl (M.D.); zak@pbs.edu.pl (S.Ż.); 2 Faculty of Chemical Technology and Engineering, West Pomeranian University of Technology in Szczecin, ul. Pułaskiego 10, 70-322 Szczecin, Poland

**Keywords:** biogas, biomethane, carbon dioxide, ceramic membranes, plasticization, polymeric membranes, pretreatment, upgrading

## Abstract

In recent years, the use of biogas as a natural gas substitute has gained great attention. Typically, in addition to methane (CH_4_), biogas contains carbon dioxide (CO_2_), as well as small amounts of impurities, e.g., hydrogen sulfide (H_2_S), nitrogen (N_2_), oxygen (O_2_) and volatile organic compounds (VOCs). One of the latest trends in biogas purification is the application of membrane processes. However, literature reports are ambiguous regarding the specific requirement for biogas pretreatment prior to its upgrading using membranes. Therefore, the main aim of the present study was to comprehensively examine and discuss the most recent achievements in the use of single-membrane separation units for biogas upgrading. Performing a literature review allowed to indicate that, in recent years, considerable progress has been made on the use of polymeric membranes for this purpose. For instance, it has been documented that the application of thin-film composite (TFC) membranes with a swollen polyamide (PA) layer ensures the successful upgrading of raw biogas and eliminates the need for its pretreatment. The importance of the performed literature review is the inference drawn that biogas enrichment performed in a single step allows to obtain upgraded biogas that could be employed for household uses. Nevertheless, this solution may not be sufficient for obtaining high-purity gas at high recovery efficiency. Hence, in order to obtain biogas that could be used for applications designed for natural gas, a membrane cascade may be required. Moreover, it has been documented that a significant number of experimental studies have been focused on the upgrading of synthetic biogas; meanwhile, the data on the raw biogas are very limited. In addition, it has been noted that, although ceramic membranes demonstrate several advantages, experimental studies on their applications in single-membrane systems have been neglected. Summarizing the literature data, it can be concluded that, in order to thoroughly evaluate the presented issue, the long-term experimental studies on the upgrading of raw biogas with the use of polymeric and ceramic membranes in pilot-scale systems are required. The presented literature review has practical implications as it would be beneficial in supporting the development of membrane processes used for biogas upgrading.

## 1. Introduction

**Figure 1:**
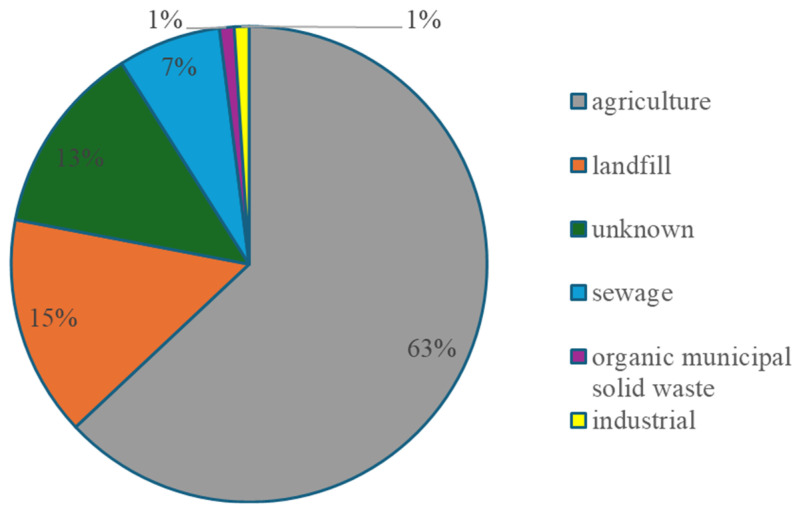
Percentage of different feedstocks in biogas production in Europe in 2020 based on data from [9].

Biogas is regarded as a renewable energy carrier that may substitute conventional energy sources. Hence, its production is well established and globally promoted. In 2022, biogas production in Europe amounted to 21 billion cubic meters (bcm) [1], and undoubtedly, there is still significant potential for further increase in biogas production. Indeed, according to the European Biogas Association data [2], it is believed that the biogas production can double by 2030. Basically, biogas is generated as a result of a biochemical conversion of organic matter via a four-step anaerobic digestion (AD) process. For this purpose, the main feedstocks used in Europe are agricultural wastewaters, landfills and sewage sludges (Figure 1). Correspondingly, it has been widely reported that AD is an energy-efficient, environmentally sustainable and marketable process for bioenergy production [3,4,5,6,7,8].

**Figure 2:**
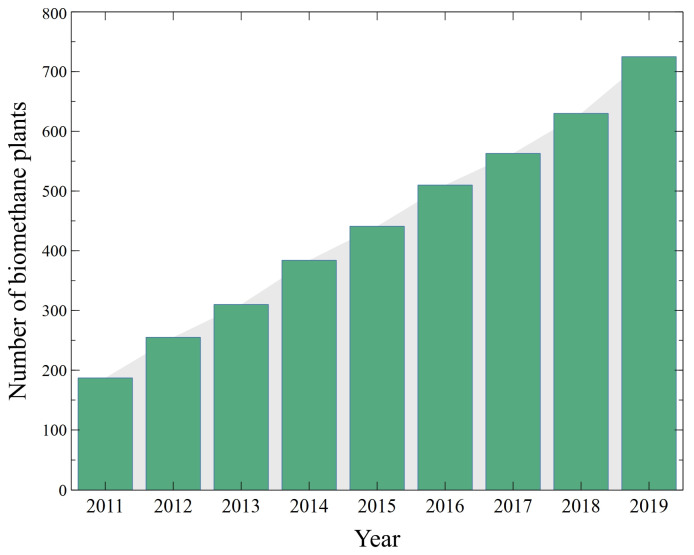
Number of biomethane plants in Europe based on data from [13].

Undoubtedly, methane (CH_4_) is the most important component of biogas. Surprisingly, biogas generated from AD is characterized by a slightly higher CH_4_ content than that produced from landfills [10]. It must be stressed that CH_4_ is a valuable source of energy as it is characterized by a higher calorific value than biodiesel, bioethanol and biomethanol [11]. Therefore, the global biogas and biomethane markets have been growing over the last years [12]. Furthermore, as displayed in Figure 2, the number of biomethane plants have systematically increased [13].

Generally speaking, biogas can be used for heat production by direct combustion, electricity production or can replace fossil fuels in the transport sector [14,15,16,17,18,19,20]. Nevertheless, the final application of biogas is determined by its composition. Although Europe is undoubtedly the world leader in terms of biogas production [21,22,23,24], it is mainly used there to generate heat and electricity [7,25]. In turn, the composition of raw biogas markedly depends on several factors, such as (I) substrate nature [26,27], (II) operational conditions [28,29] and (III) configuration of anaerobic digester [30]. Typically, in addition to CH_4_, biogas contains CO_2_, as well as small amounts of impurities, the so called ‘trace compounds’. Among them are nitrogen (N_2_), oxygen (O_2_), hydrogen (H_2_) and (H_2_S). It should be pointed out that the above-mentioned gases are undesirable and have negative effects on the performance of biogas production and plant safety [31,32]. Moreover, usually, biogas consists of volatile organic compounds (VOCs) which include, for instance, alcohols, alkanes, aromatic compounds and halogens [31,32]. As it has been indicated in [33], VOCs have no significant impact on the process performance; however, they can lead to damage to industrial installations. 

It is immediately clear that the biogas cleaning is aimed to improve the biogas quality by increasing the CH_4_ concentration. The first step, called ‘biogas purification’, is performed in order to remove impurities that are toxic, reduce the biogas heat value and lead to corrosion issues. In turn, the second step, called ‘biogas upgrading’, aims to separate CO_2_, typically down to 2% vol. Consequently, it allows to obtain biomethane with properties and a composition similar to those of natural gas [34] and meet the quality standards of natural gas grids [35]. It has been widely reported that effectively upgraded biogas, referred to as biomethane, should contain more than 95–97% CH_4_ [14,36,37,38,39,40,41]. Noteworthily, according to data presented in the International Energy Agency report [42], currently, 90% of biomethane produced worldwide is obtained by upgrading processes.

**Figure 3:**
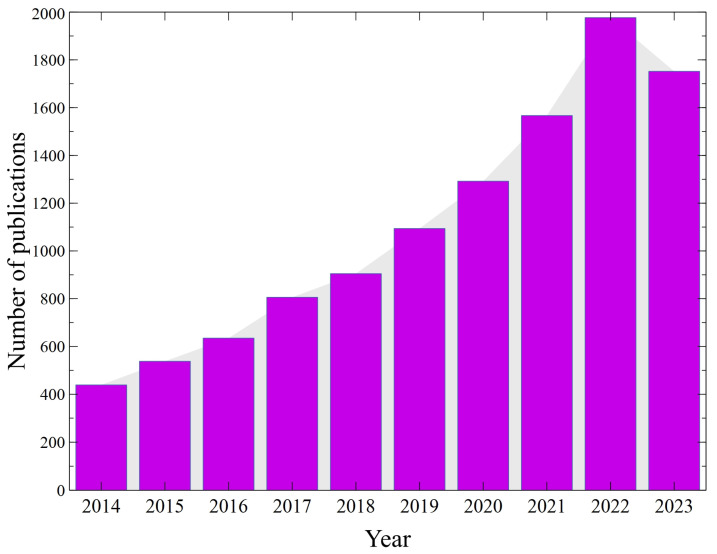
Number of articles focused on biogas upgrading according to ScienceDirect. Keywords: ‘biogas upgrading’. Data retrieved: 1 January 2024.

An important point that should be noted is that the selection of the appropriate process for this purpose is a key step that may have a significant impact on the overall technology cost. Obviously, it requires the knowledge of the characteristics of the biogas components. Hence, nowadays, significant research focus is being placed on biogas purification. Moreover, the performed literature review indicates that the number of research articles devoted to the issue of biogas upgrading has been systematically increasing over the last 10 years (Figure 3). The most remarkable result to emerge from the data is that this number has increased 4.5 times from 2014 to 2022.

It is interesting to note that there are several methods for biogas purification and upgrading. Conventional technologies include processes such as (I) water scrubbing [43,44] that shares 41% of the global upgrading market [30], (II) cryogenics [45,46], (III) chemical absorption [47,48] and (IV) swing adsorption [49,50]. Advantages and disadvantages of the above-mentioned methods have been presented in detail in several papers [3,51,52,53,54,55,56]. Moreover, Mulu et al. have demonstrated in recently published articles [57,58,59,60] that biogas purification and upgrading can also be achieved by the applications of several natural materials, such as zeolite, clay, fly ash and wood ash. Moreover, in recent years, many attempts have been made by researchers to investigate CO_2_ conversion using alternative technologies. Remarkable achievements in this field have been presented and discussed in several review articles [61,62,63].

The membrane gas separation process is a well-known technology since it was first established in the 1980s in order to remove CO_2_ from natural gas [64,65,66]. With regard to Europe, a commercial biogas upgrading installation using the gas permeation method was installed for the first time in Netherlands in 1990 [67]. As can be seen from the literature review, the separation of generated gases with the use of various membranes is of growing importance. Moreover, it is expected that the market of the membranes used for biogas upgrading will grow from USD 525.8 million in 2022 at a compound annual growth rate of 19.04% to USD 1495.91 million by 2028 [42]. It is due to the fact that this technology stands out among other methods. Indeed, membrane processes are characterized by multiple practical advantages, such as (I) high energy efficiency without generation of toxic waste, (II) small footprint due to high packing densities of membranes in modules, (III) reliability and (IV) low capital cost [3,35,52,68,69]. Moreover, as it has been indicated by Khan et al. [70], membrane processes have been shown to be a relatively straight forward. This observation is in line with that presented in [71] wherein it has been indicated that, generally, membrane plants for gas separation can be operated without supervision.

Roughly speaking, the separation of biogas with the use of membrane processes can be achieved by using a gas permeation membrane or a membrane contactor [67], which is defined as a device containing a porous membrane that separates two fluid phases (gas–liquid or liquid–liquid) [72]. Basically, the separation process is driven by a pressure difference across the membrane [73] that plays a role of a specific boundary between the permeate gas stream and the inlet gas. As a consequence, CO_2_ goes through the membrane and CH_4_ is retained [74]. The separation with the use of dense membranes is based on the solution–diffusion mechanism. It is clearly related to the affinity of molecules with the membrane material and the diffusion via the polymeric film [34]. Although this technology has several promising advantages [75,76,77], gas permeation is the most commonly used. Indeed, it has been effectively implemented on an industrial scale [64]. With the use of porous membrane, the separation mechanism is based on the difference between the sizes of molecules and membrane pores. As reported by Seong et al. [78], the recovery performance of the membrane technology is mainly defined by both the separation efficiency and the configuration design of the multi-stage membrane process. Noteworthily, the efficiency of the gas separation process is determined by the product gas purity and the gas fraction in the feed recovered with the product [79]. Importantly, according to [49], membrane technology may provide methane purity higher than 96%.

It is important to note that the appropriate design of the process depends on the further application of the upgraded biogas. Generally, biogas upgrading can be performed with the use of a single-membrane system, which consists of a membrane module, or with the use of a multi-stage process, which employs several membrane modules [80]. In the literature, there is agreement that prior to the CO_2_ removal, the biogas purification from impurities is required in order to avoid the membrane deterioration [69,71,81,82]. However, it leads to the complexity of the process variable control and the increased costs [83]. A less exhaustive solution is biogas upgrading with the use of a single-membrane separation units without pretreatment steps. It is undeniable that it is a less expensive solution and hence increases the competitiveness of membrane processes on the biogas market [69]. However, it is related to the high methane loss and CO_2_ traces [67,84]. Hence, improving the overall efficiency of this solution is an ambitious task.

Finally, to be complete, it should be pointed out that most of the information available from the literature in terms of membrane separation systems used for biogas upgrading comes from experimental studies; however, several studies have been focused on mathematical modelling as well as simulation and economic approaches [85,86,87].

In the light of the above-cited literature, the main aim of the present paper was to comprehensively examine and discuss the most recent achievements in the use of single-membrane separation units for biogas upgrading. More specifically, the present paper is in line with the conclusion presented in the recently published review article [88], wherein it has been indicated that, in the future, the enhancement in technology of biogas upgrading is expected. This suggestion, in turn, is in accordance with that presented by Kapoor et al. [89] who highlighted that although biogas upgrading is a commercially available and increasingly implemented technology, it is still not as developed as required by the biogas production sector. In this context, the importance of the presented literature review has practical implications. Indeed, the study would be beneficial in supporting the development of membrane technology used for the biogas purification.

## 2. Characteristics of the Main Biogas Impurities

The typical composition of biogas is presented in Table 1. It has been previously indicated that among the main biogas impurities are CO_2_, H_2_S, H_2_O, N_2_ and O_2_. The current section briefly presents their characteristics.

### 2.1. CO_2_

**Figure 4:**
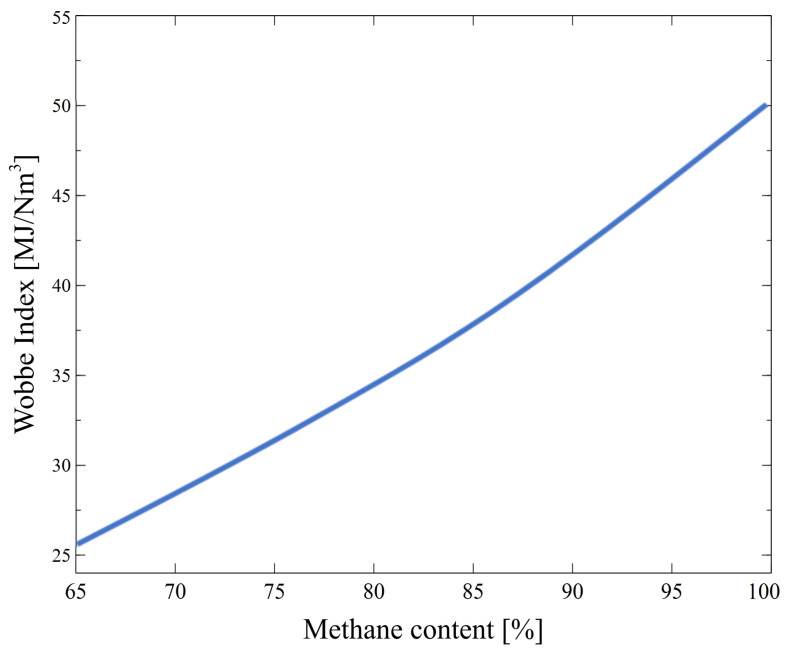
Wobbe Index as a function of CH_4_ content in biogas based on data from [111,112].

CO_2_ is a colorless gas with a molar mass of 44.01 g/mol. It is approximately 1.5 times heavier than air at ambient temperature [96,97,98,99]. It is a major contaminant in raw biogas. Indeed, typically, its content is in the range between 30 and 45 vol% (Table 1). Considering the state of research into the biogas composition, it can be clearly indicated that the CO_2_ concentration in biogas depends on several factors, such as (I) temperature, (II) pressure and (III) liquid content in the digester [41]. It is non-toxic gas; however, it decreases the calorific value of biogas, reduces its density, laminar flame speed and combustion efficiency [34,57,88]. This implies that its high content reduces the economic feasibility of direct biogas application [40] and limits its use mainly to heat and electricity generation. Furthermore, the CO_2_ leads to the corrosion of the pipeline and the wear out of the installation equipment [100,101,102,103,104]. Finally, its capture is one of the most significant technologies in biogas production [105], which allows to increase the Wobbe Index (WI) [92] (Figure 4). Generally speaking, WI is recognized as an indicator of fuel composition [106]. It is defined as the ratio of the calorific value of fuel to the square root of its specific gravity [107,108,109,110].

Consequently, an increase in the use of biogas can be achieved in a wide range of applications. On top of that, capturing CO_2_ from biogas ensures reduction in its emissions, which is equal to 57.3 t of CO_2_ per TJ of energy [113,114,115,116,117]. As a result, global warming, which may have a negative impact on the environment and human health, can be stopped. Finally, it should be pointed out that CO_2_ captured at biogas plants can be used for various industrial applications, such as the syntheses of (I) polymers, (II) urea, (III) methanol and (IV) salicylic acid [118,119,120]. Furthermore, recent studies on this topic [37,121,122,123] concluded that CO_2_ captured from biogas, in combination with H_2_, can be applied for obtaining an additional CH_4_ stream (hydrogenation process), according to the Sabatier reaction:

(1) $${CO}_{2}+{4H}_{2}\to {CH}_{4}+2H_{2}O$$

### 2.2. H_2_S

H_2_S is a colorless and flammable gas slightly heavier than air [47,96] with a molar mass of 34.08 g/mol. It is the significant impurity in the raw biogas in a concentration of up to 10,000 ppm (Table 1). Certainly, it has a negative impact on human health and is harmful to the environment [32,34,41,88,92,124,125]. A toxic concentration of H_2_S remaining in biogas is considered to be higher than 5 cm^3^/m^3^ [12,111]. Moreover, it should be noted that H_2_S is a problematic biogas compound since it is characterized by strong and peculiar odor [34,57,126,127,128]. Noteworthily, the H_2_S concentration equal to 200–300 ppm may lead to respiratory arrest [32]. In addition, it is a corrosive substance leading to the destruction of installation and piping [81,88,89,129,130,131]. For instance, the maximum allowable concentrations of H_2_S for boilers is below 1000 ppm, meanwhile for reciprocating engines, the acceptable range is below 250 ppm [49]. Its content in raw biogas depends on the percentage of proteinaceous and other sulfur compounds present in the substrate [41]. The important finding is that H_2_S concentration in biogas produced from wastewater treatment plants is generally higher than that in biogas obtained with the use of landfills as a feedstock [41,129]. The removal of H_2_S from biogas is crucial since the use of biogas as a fuel without the purification leads to the formation of sulfur dioxide (SO_2_), which is toxic to human health and has harmful environmental effects [132,133,134,135,136,137,138]. With regard to biogas, removing this impurity may have the crucial impact on the technological and economic feasibility of the upgrading process [54]. The choice of the most suitable technique for H_2_S removal from raw biogas depends on several factors. Among them are, for instance, (I) gas concentrations, (II) treatment cost and (III) H_2_S content [126].

### 2.3. H_2_O

In general, raw biogas contains saturated water vapor, with a content in the range of 1–5% (Table 1). It reduces the heating value of biogas, and in the presence of H_2_S and CO_2_, it accelerates the corrosion process [40,139]. Furthermore, it can react with H_2_S to form sulfuric acid (H_2_SO_4_) [140]. Noteworthily, Sahin and Ilbas [141] investigated the impact of H_2_O content on the biogas combustion behavior. The above-mentioned authors have demonstrated that an increase in the H_2_O content leads to a reduction in the biogas flame temperature due to the mixture dilution. In addition, the removal of H_2_O from biogas is required in order to avoid water condensation [142]. In general, the removal of water from raw biogas is conducted with the use of condenser or by the application of adsorption technologies [143].

### 2.4. N_2_ and O_2_

It is considered that the typical contents of N_2_ and O_2_ in the raw biogas are up to 15% and 3%, respectively (Table 1). It is widely accepted that, due to the anaerobic conditions, N_2_ should be absent in the reactor. Hence, its presence in the raw biogas may mean there is a denitrification issue or an air leakage in the reactor. Although N_2_ has no harmful environment effect [144,145], it leads to the decrease in the calorific value of biogas [40,92]. Likewise, the present of O_2_ in raw biogas clearly indicates that air has entered the digester. O_2_ binds hydrogen and partly binds carbon, leading to the production of compounds such as hydroxides, water and oxides [96]. Depending on the biogas temperature, the O_2_ concentration higher than 6% may lead to an explosion [142].

## 3. Application of Membranes for Biogas Upgrading

### 3.1. Membrane Types Used for Biogas Upgrading

It is well known that the worldwide use of biogas is limited. Undoubtedly, it is mainly due to its purification requirements [53]. According to the data presented in the report of IEA Bioenergy [146], the required effectiveness of the raw biogas purification depends on its future application (Table 2).

With regard to industry, for gas separation, nonporous membranes are the most commonly used [147]. With regard to material, polymeric, ceramic and composite membranes can be used. Among them, only polymeric membranes are used on an industrial scale, which is clearly related to their lower cost and the possibility to fabricate them into hollow fibers [64,148,149]. According to [52], among the most popular materials used for membranes fabrication are polyimide, polyamide (PA) and cellulose acetate (CA). Noteworthily, membranes based on CA were the first to be commercialized for biogas purification [150]. Performing the literature review indicated that the upgrading of both raw and synthetic biogas has been thoroughly investigated with the use of membranes fabricated from various polymers. Among them are mainly cellulose-based carbon [35], PI [52,151,152,153], polyetheretherketone (PEEK) [54,154], CA [64,155], polydimethylsiloxane (PDMS) [64], polyester carbonate (PEC) [69], thin-film composite polyamide (TFC PA) [82,87,156,157], polysulfone (PSf) [83] and polyethersulfone (PES) [158] membranes. It is worth noting that, with regard to gas separation, other membrane materials are also investigated. For instance, in the recently published paper [159], the separation performance of the cellulose triacetate (CTA) membrane material in humid high-H_2_S natural gas feed streams has been evaluated.

Generally, the above-mentioned membrane types are characterized by high permeability to CO_2_ and low permeability to CH_4_. As a consequence, during the biogas purification process, CO_2_ is concentrated in permeate stream, meanwhile CH_4_ is concentrated in the retentate stream. As it has been indicated in [160], the CH_4_ concentration in the retentate stream depends mainly on the following factors: (I) membrane selectivity, (II) ratio of the pressures applied on the membrane sides and (III) membrane stage-cut defined as the fraction of biogas feed that is allowed to permeate via the membrane [133,151,160,161]. An important issue that must always be considered is related to the fact that that raw biogas also contains several impurities, such as H_2_S and water vapor. Hence, it is necessary to mention that the materials used for membranes utilized in biogas upgrading should be chemically stable and resistant to these compounds [52].

Table 3 shows the ideal permeability and selectivity of selected materials for CO_2_ and CH_4_ separation reported in the literature [10,64,148,162,163].

It is important to note that polymeric membranes are stable at high operated pressures and easily scalable [83,148]. However, the most well-known limitations of this membrane type is plasticization [35], which is the swelling of the membrane structure and has come to be used to refer a ‘phenomenon, caused by the dissolution of certain substances in the polymeric matrix’ [64]. In general, it leads to an increase in the fractional free volume of the membrane [164,165,166,167]. As a consequence, a permeability of CO_2_ increases, and finally, a decrease in the membrane selectivity is observed [82,148,168]. CO_2_ is the most significant impurity present in biogas affecting this phenomenon; however, water vapor and trace components (e.g., siloxanes, hydrocarbons) may also have a significant impact [151].

Membranes can be classified as hollow-fiber, spiral-wound and enveloped membranes. According to Pak et al. [155], for gas separation, hollow fiber membranes are the most popular. The above-mentioned authors have indicated that it is related to the fact that they have several significant advantages, such as (I) high flexibility, (II) large area to unit volume ratio and (III) high productivity. Noteworthily, Chmielewski et al. [151] have indicated that asymmetric hollow-fiber modules may have a three times larger area per unit volume compared to spiral-wound ones. These findings are in line with those presented in the current study. Indeed, performing the literature review allowed to demonstrate that, in most of the studies aimed to investigate the upgrading of both synthetic and raw biogas, the hollow-fiber membranes have been used (Table 4).

In turn, ceramic membranes are characterized by unique advantages, such as excellent resistance as well as thermal and mechanical stability. It is equally important that they exhibit a longer service as well as provide higher selectivity and permeability than polymeric ones; nevertheless, they are more expensive [148,149,169,170,171,172,173,174]. The performed literature review allows to demonstrate that experimental studies on their application in this field have been neglected. Indeed, to the best of the authors’ knowledge, the open-access literature contains no experimental studies investigating the application of ceramic membranes in single-membrane systems for biogas upgrading. It is essential to mention that this finding is in line with that presented in [175], wherein it has been indicated that ceramic membranes for gas separation are still in an early technological stage. Taking the above-mentioned into account, it can be concluded that further studies are needed to investigate the efficiency of ceramic membranes in biogas enrichment.

**Figure 5:**
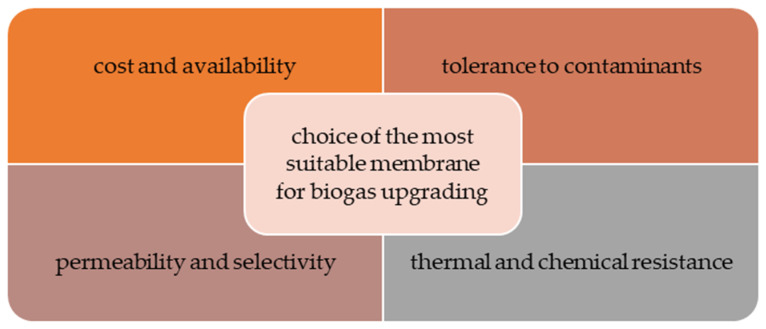
Factors affecting the choice of the most suitable membrane for biogas upgrading.

Finally, it should be emphasized that the choice of the most suitable membrane for biogas separation is a great challenge. It is related to the fact that it depends on several factors. Among them, for instance, are (I) membrane cost and material availability, (II) tolerance to impurities present in biogas, (III) thermal and chemical resistance and (iv) fundamental parameters defining membrane separation performance: permeability and selectivity [52,148,176] (Figure 5). Clearly, the permeability is equal to the product of gas solubility and membrane diffusivity [177]. In turn, the membrane selectivity α describes its ability to separate two gases, A and B, and it is defined as the ratio of permeability coefficients *p_A_* and *p_B_* and is as follows [147,178,179,180]:

(2) $$\alpha_{A/B}=\frac{p_{A}}{p_{B}}$$

Permeability coefficients indicate the rate at which gas molecules are transported through the membrane [181].

### 3.2. Upgrading of Synthetic Biogas

Performing the literature review allows for demonstrating that the applications of single-membrane permeation systems have been investigated for synthetic biogas characterized by CH_4_ content in the range from 50 to 90 mol% (Table 4). Noteworthily, in several studies [35,54,152,156], the H_2_S present in the gas was considered. However, it has been found that most of the studies have been performed in order to determine membrane applications for short-term processes. Meanwhile, the development of membrane processes used for biogas upgrading requires the investigations on long-term stability and durability of membranes used for this purpose.

Sedláková et al. [156] have thoroughly investigated the removal of CO_2_ and H_2_S from synthetic biogas. For this purpose, thin-film composite (TFC) membranes with PA skin layer have been used. The authors have clearly indicated that the application of the above-mentioned membrane type has a significant advantage. Indeed, due to the fact that membranes show the good ability to work in a humid environment, the pretreatment of gas from water vapor is not required. At the same time, it has been demonstrated that the use of membranes for 120 h allowed for maintaining the performance of the membranes. It becomes apparent from the discussed study that the application of this membrane type ensured the effective removal of H_2_S and CO_2_ from synthetic biogas in a single step. However, their successful separation requires relative humidity of feed above 90%. The process allowed for obtaining the CH_4_ concentration in the retentate stream of up to 99 mol%.

The application of TFC membranes with PA skin layer for the upgrading of synthetic biogas has also been documented in [82,87]. However, in the above-mentioned studies, experimental investigations have been performed for biogas free of H_2_S. For instance, Wojnarova et al. [82] investigated the applicability of the membrane on pilot-scale systems. It has been demonstrated that a spiral-wound membrane module based on TFC membrane allowed to increase the CH_4_ content from 52 vol% in the feed to about 95 vol% in the retentate stream. Over the entire separation process, the obtained methane recovery ranged between 46.4 and 49.9%, with an average value equal to 48.24%.

Several researchers have made remarkable achievements in the investigation of the application of hollow-fiber PA membranes for the upgrading of synthetic biogas [151,152,153]. For instance, Harasimowicz et al. [152] have shown that, for this purpose, multi-stage systems including special gas pretreatment are not required. Indeed, the used membranes demonstrated a high permeability to common impurities present in biogas, such as H_2_O and H_2_S. In addition, the above-mentioned study demonstrated that a single-stage unit ensures the achievement of 77.4% CH_4_ recovery. The above-discussed results are in agreement with those obtained in [153], wherein it has been documented that a hollow-fiber PA membrane used for upgrading the model gas (80 vol% CH_4_ and 20 vol% CO_2_) allowed to obtain a retentate with 93.8 vol% of CH_4_.

Preliminaries experimental test presented in [154] have demonstrated a feasibility of integrating anaerobic digestion plant with PEEK hollow-fiber membranes in terms of biomethane production. With regard to the impact of biogas impurities on the membrane selectivity and permeability, in study [54], it has been documented that the presence of H_2_S does not have any impact on the selectivity of the PEEK hollow-fiber membranes. In turn, Brunetti et al. [35] in a recently published paper have demonstrated that the H_2_S present in synthetic biogas led to a reduction in the permeability of cellulose-based carbon hollow-fiber membranes in terms of both CO_2_ and CH_4_ by 43% and 25%, respectively. In addition, it has been noted that humidified gas streams caused a decrease in the CO_2_ permeability of about 67%. However, for more than 180 days of the process run, the membranes used in the above-mentioned study exhibited a remarkable CO_2_/CH_4_ selectivity.

In turn, Pak et al. [155] have performed separation tests in order to verify the separation performance of CA asymmetric hollow-fiber membranes, which have been prepared through a dry/wet spinning process. For this purpose, a binary gas (CO_2_/CH_4_ 60:40) was used. Results presented in the above-mentioned study showed that this type of membrane allows the obtainment of methane with a purity higher than 97% and a recovery efficiency equal to 77% in a single-stage permeation. The above-mentioned authors have indicated that the single-stage process may not be sufficient for recovering high-purity gas at a high recovery efficiency. In a later published study, Cerveira et al. [64] in order to attain CO_2_ removal investigated the application of a composite commercial cellulose acetate membrane and a dense film of PDMS. For this purpose, gases mixture with a molar composition of 50% CH_4_ and 50% CO_2_ have been used. It has been clearly documented that CA membrane was characterized by the higher CO_2_/CH_4_ selectivity compared to the PDMS one. Consequently, a CH_4_ recovery for the CA and PDMS membranes was equal to 86.8% and 19.8%, respectively. This finding can be attributed to the molecular structures of polymers. Indeed, glassy polymeric membranes, such as made of CA, are more selective towards the size and shape of gas molecules compared to rubbery ones, including PDMS ones. Indeed, glassy polymers are characterized by densely packed polymer chains that have restricted mobility. Contrarily, rubbery polymeric membranes are flexible and may provide more fractional free volume, resulting in decreased membrane selectivity [182,183,184,185].

To sum up, the conclusion can be drawn that most of the available experimental studies reported in the literature have been conducted with the use of laboratory-scale membrane systems. Hence, it can be concluded that further experimental investigations are needed to study the application of pilot-scale single systems for synthetic biogas upgrading.

### 3.3. Upgrading of Raw Biogas

Performing the literature review allows to indicate that experimental studies focused on the upgrading of the raw biogas by single-membrane permeation systems are quite limited (Table 4). More specifically, investigations have been carried out with the use of both laboratory- and pilot-scale systems. Moreover, although studies on process stability and long-term durability of membranes are key aspects for industrial applications, most of the experiments reported in the literature were short-term.

Results presented in [52] are of great importance for the design of membrane separation units for biogas upgrading. In the above-mentioned study on upgrading real biogas from the anaerobic fermentation of sewage sludge, the polyimide fiber membranes have been used. As a matter of fact, membrane separation was tested on a pilot scale. The obtained results have demonstrated that the performed process allowed to achieve a CH_4_ content higher than 95 vol% in the produced biomethane. This noteworthy finding indicated that the membrane separation unit used in the discussed study can be successfully used for the upgrading of biogas. Indeed, it allows to obtain biogas characterized by a concentration of H_2_S of up to 100 mg/m^3^ and a relative humidity at a level of 40−50%. Hence, it has been recommended for biogas units in wastewater treatment plants.

In turn, the application of polyimide hollow-fiber module for the purification of biogas from agricultural plant has been investigated by Chmielewski et al. [151]. It has been noted that the hollow-fiber PA membranes used in the above-mentioned study are efficient. Indeed, they demonstrated a high selectivity for separating CH_4_ from CO_2_, H_2_S and H_2_O. More specifically, performing the membrane process allowed to obtain the retentate characterized by a high methane concentration (of up to 90% volume). In addition, it was free of H_2_S, which was recirculated to the hydrolyzer in order to achieve an O_2_-free atmosphere. On the other hand, the permeate contained less than 5 vol.% of CH_4_, which indicated that the membranes ensured low losses of this biogas component. Finally, the above-mentioned authors have pointed out that the upgraded biogas could be employed for household uses.

Nemestóthy et al. [153] have demonstrated the results of a long-term biogas upgrading process with the use of hollow-fiber PA membranes. The tested real gaseous mixtures contained CH_4_, CO_2_, N_2_ and unknown trace substances. It has been reported that the membranes used in the above-mentioned study allowed to increase the CH_4_ concentration in biogas from 57.4 to 81.7 vol% in the retentate. As a matter of fact, the steady level of CH_4_ recovery was equal to 82.9%. Moreover, it should be pointed out that the performed experiments revealed the adequate time stability of membrane purification. Hence, the above-mentioned authors have indicated that the application of this membrane type is worthy of further investigation under industrial conditions in the field.

Stern et al. [160] have investigated the performance of a bench-scale membrane pilot plant for biogas upgrading in a municipal wastewater treatment plant. For this purpose, hollow-fiber membranes with unknown polymeric material have been used. In order to prevent the condensation of organic impurities in the system, the biogas pretreatment was conducted by heat exchange and a slight feed heating. It has been documented that the application of a bench-scale membrane pilot allows to increase the CH_4_ concentration from 62–63 to 97 mol%. However, it has been found that the used membranes cannot be successfully applied for reducing the H_2_S concentration injected in the raw biogas. Indeed, the H_2_S concentration decreases from 0.5 mol% to about 0.2 mol%. Hence, the above-mentioned authors have indicated that, for this purpose, it is recommended to apply two different types of membranes systems, characterized by high CO_2_/CH_4_ and H_2_S/CH_4_ selectivities, respectively.

Efficient raw biogas upgrading to biomethane quality with the use of thin asymmetric non-porous hollow-fiber polyester carbonate membranes has been presented in study [69]. The authors have documented that the used membranes are able to operate in the presence of humidity and sulfur species present in biogas. Moreover, it has been clearly demonstrated that there is a possibility of having a membrane operation without any pretreatment steps for removing of contaminants in biogas from the agricultural plant. More specifically, the application of the single-stage configuration allowed to obtain 96 mol% purity of CH_4_ in the permeate. Hence, membrane separation is undoubtedly competitive with other known methods used for biogas upgrading. Indeed, the authors have pointed out that it allows to obtain the methane recovery with a decrease in the investment expenditure of approximately 20%. To sum up, it should be pointed out that the use of polyester carbonate hollow-fiber membranes is a promising method for a wide application in gas separations, and it is worth investigating further.

In a follow up study [82], the application of a swollen TFC polyamide membrane for the upgrading of raw biogas obtained from the first digestion stage of an agricultural plant has been demonstrated. As it has been mentioned in the Introduction Section, it is generally accepted that biogas purification from impurities is required in order to avoid membrane deportation. On the other hand, according to the discussed study, TFC membranes used extensively for reverse osmosis desalination do not require a biogas pretreatment to remove water vapor as well as other impurities such as hydrogen sulfide and ammonia. In addition, it has been documented that the used membranes ensured an increase in CH_4_ from 52 vol% in the feed to 98 vol% in the retentate stream. Moreover, it allowed to achieve H_2_S concentration in the retentate at the level of 10 ppm. Similar results have been obtained in [157], wherein it has been shown that a reverse osmotic thin-film composite membrane with a swollen PI layer allows to increase CH_4_ content from 62.5% in raw biogas to 95% in the retentate.

As it has been mentioned above, studies focused on the application of membrane systems for the upgrading of raw biogas are very limited. Hence, it should be pointed out that further experimental investigations are needed to determine the effectiveness of such systems for the upgrading of real biogas under various operational parameters. This conclusion is supported by the fact that the separation of synthetic and raw biogas should be considered differently. It is due to the differences in the framework of designing membrane systems for such purposes. In addition, it is highly recommended to perform long-term experimental studies with the use of pilot-scale membrane installations, which is necessary from the technological point of view.

## 4. Conclusions and Further Challenges

It is well known that the worldwide use of biogas is limited mainly due to its purification requirements. For this purpose, membrane systems can be successfully applied. Indeed, many researchers have reported that membrane technology is suitable to replace conventional technologies. In addition, biogas upgrading with the use of membranes without pretreatment steps increases the competitiveness of this technology on the biogas market. Hence, the main aim of this review was to comprehensively examine and discuss the most recent achievements in the use of single-membrane separation units for biogas upgrading.

It has been clearly demonstrated, in recent years, that considerable progress has been made with the use of polymeric membranes for this purpose. For instance, it has been documented that the application of thin-film composite membranes with a swollen polyamide layer ensures the successful upgrading of raw biogas and eliminates the need for its pretreatment. The importance of the performed literature review is that the biogas enrichment in a single step allows to obtain upgraded biogas that could be employed for household uses. Nevertheless, this solution may not be sufficient for obtaining high-purity gas at high recovery efficiency. Hence, in order to obtain biogas that could be used for applications designed for natural gas, a membrane cascade may be required.

However, most of the studies available in the literature have been conducted on synthetic biogas; meanwhile, the data on the raw biogas are very limited and have not been dealt with in depth. Finally, it has been noted that most of the studies have been performed with the use of laboratory-scale membrane systems.

The evidence from this study implies that in order to thoroughly evaluate the possibility of raw biogas upgrading with the use of membrane technology, the further experimental studies are required. Although ceramic membranes demonstrate several advantages, to the best of the authors’ knowledge, the open-access literature contains no experimental study investigating their application in this field. Hence, the studies on biogas upgrading with the use of ceramic membranes in single-membrane systems are required. It is important to note that the recommended specific areas of future research also include studies aimed at examining the long-term stability and durability of various membranes under industrial conditions. It is due to the fact that long-term investigations are a key aspect for industrial applications.

Finally, the importance of the presented literature review has practical implications as it would be beneficial in supporting the development of membrane processes used for biogas upgrading.

## Figures and Tables

**Table 1:** Biogas composition from AD reported in the literature [6,43,90,91,92,93,94,95].

Compound Formula	Unit	Value
CH_4_	vol%	55–70
CO_2_	vol%	30–45
H_2_S	ppm	0–10,000
H_2_0	vol%	1–5
N_2_	vol%	0–15
O_2_	vol%	0–3
H_2_	vol%	0–1
NH_3_	ppm	0–100

**Table 2:** Requirements to remove biogas impurities based on data from [146].

Biogas Application	H_2_S	CO_2_	H_2_O
gas heater	required, concentration lower than 1000 ppm	not required	not required
kitchen stove	required	not required	not required
stationary engine	required, concentration lower than 1000 ppm	not required	no condensation required
natural gas grid	required	required	required
vehicle fuel	required	recommended	required

**Table 3:** Ideal permeability and selectivity of selected materials reported in the literature [10,64,148,162,163].

Membrane Material	CO_2_ Permeability at 30 °C [Barrer]	CH_4_ Permeability at 30 °C [Barrer]	Selectivity CO_2_/CH_4_
cellulose acetate (CA)	6.30	0.21	30.0
polyimide (PI)	10.70	0.25	42.8
polysulfone (PSf)	5.60	0.25	6.89
polydimethylsiloxane (PDMS)	2700	800	3.38

**Table 4:** Single-membrane permeation systems for upgrading of synthetic and raw biogas based on literature data.

Biogas	System Scale	Membrane				Operation Conditions				Feed Content			Permeate Content			Retentate Content			CH_4_ Recovery [%]	Ref.
		Manufacturer	Module	Material	Area [m^2^]	T [K]	Feed Pressure [Bar]	Permeate Pressure [Bar]	Feed Flow Rate	CH_4_	CO_2_	H_2_S	CH_4_	CO_2_	H_2_S	CH_4_	CO_2_	H_2_S		
synthetic	laboratory	-	hollow fiber	cellulose-based carbon	0.0009	308	9.6	1.03–1.20	300–500 mL(STP)/min	60.2 mol%	39.8 mol%	-	N.A.	N.A.	-	N.A.	N.A.	-	N.A.	[35]
synthetic	laboratory	-	hollow fiber	cellulose-based carbon	0.0009	308	9.6	1.03–1.20	300–500 mL/min	56.9 mol%	37.3 mol%	203 ppm	N.A.	N.A.	N.A.	N.A.	N.A.	N.A.	N.A.	[35]
synthetic	N.A.	PoroGen Corp. (Woburn, MA, USA)	hollow fiber	PEEK	N.A.	N.A.	3.9–7.8	0.2–0.4	25.5–41.0 kg/h	53.5 vol%	40.2 vol%	0.2 vol%	N.A.	N.A.	0.01–0.16 vol%	N.A.	N.A.	0.05–0.22 vol%	65.0–71.0	[54]
synthetic	laboratory	-	spiral wound	CA	0.0010	298	6.0; 11.0 and 16.0	N.A.	N.A.	50.0 mol%	50.0 mol%	-	N.A.	N.A.	-	N.A.	N.A.	-	86.8	[64]
synthetic	laboratory	-	hollow fiber	PDMS	0.0010	298	6.0 and 16.0	N.A.	N.A.	50.0 mol%	50.0 mol%	-	N.A.	N.A.	-	N.A.	N.A.	-	19.8	[64]
synthetic	pilot	DuPont-Filmtec (Edina, MN, USA)	spiral wound	TFC PA	1.2100	293	3.0	N.A.	0.46–0.50 L/min	52.0 vol%	48.0 vol%	-	N.A.	N.A.	-	94.3–95.8 vol%	~1.5–7.0 vol% ^1^	-	48.2	[82]
synthetic	laboratory	-	hollow fiber	PSf	N.A.	293	2.0–20.0	N.A.	N.A.	65.0 vol%	35.0 vol%	-	N.A.	N.A.	-	N.A.	N.A.	-	N.A.	[83]
synthetic	laboratory	Toray Membrane USA, Inc. (Poway, CA, USA)	N.A.	TFC PA	0.0125	294	0.7–1.2	N.A.	32 mL(STP)/min	53.7 mol%	46.3 mol%	-	15.5 mol%	44.9 mol%	-	79.6 mol%	20.5–mol%	-	N.A.	[87]
synthetic	laboratory	Koch Membrane Systems, Inc. (Wilmington, DE, USA)	N.A.	TFC PA	0.0125	294	2.5–4.5	N.A.	30 mL(STP)/min	90.0 mol%	10.0 mol%	-	1.6 mol%	3.5 mol%	-	91.3 mol%	8.7 mol%	-	N.A.	[87]
synthetic	laboratory	UBE Europe GmbH (Düsseldorf, Germany)	hollow fiber	PI	0.1800	N.A.	2.0–8.0	N.A.	10–1200 Nl/h	50.0–80.0 vol%	20.0–50.0 vol%	-	~10.0 vol% ^1^	˂5%	-	up to 90.0 vol%	N.A.	-	N.A.	[151]
synthetic	bench	UBE Europe GmbH (Düsseldorf, Germany)	hollow fiber	PI	N.A.	313	6.0	0	100 N dm^3^/h	68.0 mol%	30.0 mol%	2 mol%	35.7 mol%	61.0 mol%	3.35 mol%	93.5 mol%	5.7 mol%	0.95 mol%	N.A.	[152]
synthetic	laboratory	Ube Industries, Ltd. (Düsseldorf, Germany)	hollow fiber	PI	N.A.	303	7.0–14.5	N.A.	N.A.	80.0 vol%	20.0 vol%	-	53.2 vol%	46.8 vol%	-	93.8 vol%	6.2 vol%	-	72.7–90.8	[153]
synthetic	N.A.	PoroGen Corp. (Woburn, MA, USA)	hollow fiber	PEEK	18.5800	298	3.0–20.0	N.A.	18–96 kg/h	54.4 vol%	45.6 vol%	-	N.A.	N.A.	-	~97.0 vol% ^1^	N.A.	-	40.0–85.0 ^1^	[154]
synthetic	N.A.	PoroGen Corp. (Woburn, MA, USA)	hollow fiber	PEEK	18.5800	298	3.0–20.0	N.A.	18–96 kg/h	60.0 vol%	40 vol%	-	N.A.	N.A.	-	~100 vol% ^1^	N.A.	-	25.0–90.0 ^1^	[154]
synthetic	laboratory	-	hollow fiber	CA	0.1800	room	3.0	N.A.	2.4 cc/min	60.0 mol%	40.0 mol%	-	N.A.	N.A.	-	>97.0 mol%	N.A.	-	77.0	[155]
synthetic	laboratory	Toray Membrane USA, Inc. (Poway, CA, USA)	spiral wound	TFC PA	0.1246	287-296	4.0–5.0	N.A.	14–100 mL(STP)/min	56.1 mol%	43.8 mol%	1155 ppm	36.1 mol%	63.7 mol%	1362 ppm	99.0	1.0 mol%	3 ppm	N.A.	[156]
raw	pilot	Ube Industries, Ltd. (Düsseldorf, Germany)	hollow fiber	PI	N.A.	288-298	6.0–8.0	N.A.	7 m^3^/h	61.8 vol%	37.9 vol%	100 mg/m^3^	25.2 vol%	74.9 vol%	72.86 mg/m^3^	96.4 vol%	2.2 vol%	21.25 mg/m^3^	N.A.	[52]
raw	laboratory	Generon (Houston, TX, USA)	hollow fiber	PEC	0.0110	308	7.0	19.9 m^3^/h	54 m^3^/h	51.0 mol%	48.0 mol%	0.09 mol%	96 mol%	3 mol%	0.07 mol%	24.0 mol%	74 mol%	0.1 mol%	69.4	[69]
raw	pilot	Dupont Dow Filmtec (Edina, MN, USA)	spiral wound	TFC PA	1.2100	293	3.0	N.A.	0.861–1.072 L/min	52.5 vol%	42.8 vol%	55 ppm	N.A.	N.A.	N.A.	97.0 vol%	0.9 vol%	5 ppm	46.9–49.1	[82]
raw	pilot	N.A.	hollow fiber	PI	0.1800	N.A.	2.0–90.0	N.A.	100 Nl/h	69.0 vol%	30.0 vol%	20 ppm	~3.5 vol% ^1^	˂5%	N.A.	up to 90.0 vol%	N.A.	-	N.A.	[151]
raw	laboratory	Ube Industries, Ltd. (Düsseldorf, Germany)	hollow fiber	PI	N.A.	303	4.3–8.5	N.A.	N.A.	70.0 vol%	19.8 vol%	N.A.	49.3 vol%	42.8 vol%	N.A.	80.7 vol%	7.5 vol%	N.A.	76.0–94.3	[153]
raw	industrial	Ube Industries, Ltd. (Düsseldorf, Germany)	hollow fiber	PI	N.A.	303	10.8	N.A.	N.A.	57.4 vol%	39.0 vol%	N.A.	21.6 vol%	75.8 vol%	N.A.	81.7 vol%	14.6 vol%		N.A.	[153]
raw	N.A.	Koch Membrane System Inc. (Wilmington, DE, USA)	flat sheet	TFC PA	N.A.	294	2.0–5.0	N.A.	13.5 mL/min	62.5 vol%	35.5 vol%	N.A.	N.A.	N.A.	N.A.	95.0 vol%	N.A.	N.A.	N.A.	[157]
raw	bench	N.A.	hollow fiber	N.A.	0.9300	305	36.0 and 29.0	N.A.	2.4∙10^−4^ –2.8∙10^−4^ m^3^/s and 1.7∙10^−4^ –1.9∙10^−4^ m^3^/s	62.0–63.0 mol%	36.5–37.5 mol%	~0.5 mol%	N.A.	16.0–21.0 mol%	N.A.	97.0 mol%	N.A.	N.A.	83.0	[160]

^1^ Data from a figure. CA—cellulose acetate; PDMS—polydimethylsiloxane; PEC—polyester carbonate; PEEK—polyetheretherketone; PES—polyethersulfone; PA—polyamide; PI—polyimide; PIM-TMN-Trip—ultrapermeable benzotriptycene-based polymer of intrinsic microporosity; PPSU—polyphenylsulfone; PSf—polysulfone; TFC—thin-film composite; and N.A.—not available.

## Data Availability

The data presented in this study are available on request from the corresponding author. The data are not publicly available due to the institutional repository being under construction.
